# From Plastics to Prognosis: *ANO4* Susceptibility Links Phthalate Exposure to Prostate Cancer Progression

**DOI:** 10.3390/diagnostics16050794

**Published:** 2026-03-07

**Authors:** Chi-Fen Chang, Shu-Pin Huang, Yei-Tsung Chen, Lih-Chyang Chen, Chao-Yuan Huang, Chia-Cheng Yu, Victor C. Lin, Te-Ling Lu, Bo-Ying Bao

**Affiliations:** 1 Department of Anatomy, School of Medicine, China Medical University, Taichung 406, Taiwan; cfchang@mail.cmu.edu.tw; 2 Graduate Institute of Clinical Medicine, College of Medicine, Kaohsiung Medical University, Kaohsiung 807, Taiwan; shpihu@kmu.edu.tw; 3 Department of Urology, Kaohsiung Medical University Hospital, Kaohsiung 807, Taiwan; 4 Institute of Medical Science and Technology, College of Medicine, National Sun Yat-sen University, Kaohsiung 804, Taiwan; 5 Department of Life Sciences and Institute of Genome Sciences, National Yang Ming Chiao Tung University, Taipei 112, Taiwan; yeitsungchen@ym.edu.tw; 6 Department of Medicine, Mackay Medical College, New Taipei City 252, Taiwan; lihchyang@mmc.edu.tw; 7 Department of Urology, National Taiwan University Hospital, College of Medicine, National Taiwan University, Taipei 100, Taiwan; cyh0909@ntu.edu.tw; 8 Division of Urology, Department of Surgery, Kaohsiung Veterans General Hospital, Kaohsiung 813, Taiwan; ccyu@vghks.gov.tw; 9 Department of Urology, School of Medicine, National Yang-Ming University, Taipei 112, Taiwan; 10 Department of Pharmacy, College of Pharmacy and Health Care, Tajen University, Pingtung 907, Taiwan; 11 Department of Urology, E-Da Hospital, Kaohsiung 824, Taiwan; ed102161@edah.org.tw; 12 School of Medicine for International Students, I-Shou University, Kaohsiung 840, Taiwan; 13 Department of Pharmacy, China Medical University, Taichung 404, Taiwan; lutl@mail.cmu.edu.tw

**Keywords:** plasticizer, phthalate, prostate cancer, gene–environment interaction, ANO4, pathway analysis

## Abstract

**Background/Objective:** Di-2-ethylhexyl phthalate and its bioactive metabolite mono-2-ethylhexyl phthalate (MEHP) are ubiquitous endocrine-disrupting chemicals implicated in carcinogenesis. However, the molecular mechanisms linking MEHP exposure, host genetic susceptibility, and prostate cancer progression remain incompletely defined. **Methods:** We integrated transcriptomic profiling of MEHP-exposed human prostate epithelial cells with a genetic association study of 630 patients with prostate cancer receiving androgen deprivation therapy. MEHP-responsive genes were identified from public microarray datasets and subjected to pathway enrichment analyses. Germline single-nucleotide polymorphisms (SNPs) in MEHP-regulated genes were evaluated for their association with progression-free survival, overall survival, and cancer-specific survival. The clinical and functional relevance of the key genes was further assessed using large-scale public prostate cancer expression datasets. **Results:** MEHP exposure induced widespread transcriptional reprogramming, prominently suppressing focal adhesion and cell–matrix interaction pathways. Genetic analyses identified multiple prognostically relevant SNPs within MEHP-responsive genes, with anoctamin 4 (*ANO4*) variants showing consistent associations across all clinical endpoints. The minor allele of rs17485225 in *ANO4* was significantly associated with reduced all-cause and prostate cancer-specific mortality. Pooled analyses revealed reduced *ANO4* expression levels in prostate cancer tissues and improved survival in patients with high *ANO4* expression levels. Pathway analyses linked low *ANO4* expression levels with enhanced cell cycle activity and compromised cell adhesion. **Conclusions:** Our findings suggest that *ANO4* may act as a mediator of MEHP-associated prostate cancer progression and support a gene–environment interaction model in which environmental toxicant exposure and germline variation converge on focal adhesion dysregulation to potentially contribute to aggressive disease.

## 1. Introduction

Di-2-ethylhexyl phthalate (DEHP) is one of the most extensively used plasticizers globally. It is incorporated into polyvinyl chloride-based materials to enhance their flexibility and durability. Because of its non-covalent incorporation into plastics, DEHP readily leaches into the surrounding environment and is ubiquitously detected in food packaging, medical devices, and consumer products, resulting in widespread human exposure via ingestion, inhalation, dermal contact, and parenteral routes during medical procedures [1]. Following exposure, DEHP is rapidly hydrolyzed to its primary metabolite, mono-2-ethylhexyl phthalate (MEHP), a major biologically active and toxicologically relevant species in human tissues. MEHP is consistently detected in human urine and blood, and it disrupts endocrine, metabolic, and cellular homeostasis [2]. Consequently, DEHP and MEHP are classified as endocrine-disrupting chemicals of significant public health concern, and accumulating evidence links their exposure to reproductive abnormalities, metabolic dysfunction, and carcinogenesis [3].

An expanding body of experimental evidence indicates that MEHP exerts biologically plausible cancer-promoting effects across multiple organ systems. Several recent studies have implicated MEHP in the initiation and progression of prostate cancer. In vitro and in vivo prostate cancer models have demonstrated that MEHP exposure enhances cellular proliferation, migration, and invasion, whereas chronic exposure increases tumorigenicity and reprograms signaling pathways central to malignant progression, including phosphatidylinositol-3-kinase/AKT and mitogen-activated protein kinase pathways and metabolic regulatory networks [4]. Mechanistically, MEHP functions as an endocrine-disrupting ligand capable of modulating nuclear receptor activity, most notably that of peroxisome proliferator-activated receptor (PPAR) family members, and altering androgen and steroid hormone signaling. Simultaneously, MEHP induces oxidative stress, mitochondrial dysfunction, and inflammasome activation, which converge to promote chronic inflammation, cellular survival, and genomic instability [5,6]. Epigenetic dysregulation represents an additional and increasingly recognized mechanism of MEHP action, and it has been associated with alterations in DNA methylation landscapes and downstream gene expression programs relevant to oncogenesis and tumor progression [7,8]. Although epidemiological data directly linking phthalate metabolites to prostate cancer remain limited, population-based studies have reported associations between phthalate exposure and the risk of several malignancies, as well as intermediate biomarkers of proliferative potential, such as leukocyte telomere length, supporting biological plausibility in humans [9,10]. Importantly, inter-individual variability in genes involved in DEHP/MEHP metabolism (including carboxylesterases, UDP-glucuronosyltransferases, and sulfotransferases) and MEHP-responsive signaling pathways may influence internal exposure, receptor sensitivity, and downstream transcriptional responses. While studies specifically examining MEHP–gene interactions in prostate cancer are scarce, prior investigations have demonstrated that polymorphisms in PPAR genes and xenobiotic-metabolizing enzymes can modify the associations between phthalate exposure and cancer- or hormone-related outcomes, supporting a credible gene–environment interaction framework [11,12,13].

Understanding the interplay among MEHP exposure, host genetic susceptibility, and prostate cancer progression is particularly relevant for patients receiving androgen deprivation therapy (ADT), in which hormonal perturbations may intersect with endocrine-disrupting environmental exposure. The identification of germline variants within MEHP-regulated pathways that confer an increased risk of aggressive disease or adverse outcomes may reveal novel biomarkers and mechanistic insights into environmental carcinogen–host genome interactions. Accordingly, the present study evaluated the association between variants of MEHP-regulated genes and prostate cancer progression in a cohort of 630 patients treated with ADT.

## 2. Patient and Methods

### 2.1. Transcriptomic Analysis of MEHP Exposure in Human Prostate Epithelial Cells

To characterize the transcriptional responses induced by MEHP, publicly available gene expression datasets, including GSE67396 and GSE67397, were obtained from the Gene Expression Omnibus. These datasets were generated using the immortalized non-tumorigenic human prostate epithelial cell line, PNT1A. Cells were treated for 48 h with a vehicle control (0.04% dimethyl sulfoxide) or low (0.01 μM), medium (1 μM), or high (100 μM) concentrations of MEHP. Transcriptomic profiling was performed using the Agilent SurePrint G3 Human GE 8 × 60 K microarray platform (Agilent, Santa Clara, CA, USA). The GSE67396 dataset comprised four control samples, three low-dose samples, and four medium-dose samples, whereas GSE67397 included four control samples and four high-dose samples. Processed expression matrices were downloaded and analyzed using R (v4.5.1; R Foundation for Statistical Computing, Vienna, Austria). Data normalization and precision weighting were performed using the Voom function in the Limma package (v3.64.1). Differential gene expression analyses comparing MEHP-treated samples with vehicle controls were conducted using Limma. To preserve biological signals for downstream pathway analyses in this exploratory toxicogenomic setting, genes were initially filtered at a nominal *p*-value < 0.05, followed by a stringent requirement for consistent directional expression across multiple MEHP concentrations. Gene set enrichment analysis (GSEA) was performed using ranked log fold change values to identify significantly altered biological processes and pathways [14]. Enrichment analyses were conducted for Gene Ontology (GO) categories (biological processes, molecular functions, and cellular components) and Kyoto Encyclopedia of Genes and Genomes (KEGG) pathways using the clusterProfiler package (v4.16.0).

### 2.2. Study Population and Clinical Data Collection

The clinical cohort was derived from an ongoing hospital-based prostate cancer study conducted in Taiwan [15]. Patients with histologically confirmed prostatic adenocarcinoma were recruited from three medical centers: National Taiwan University Hospital, Kaohsiung Medical University Hospital, and Kaohsiung Veterans General Hospital. Eligible patients received ADT, including bilateral orchiectomy, or luteinizing hormone–releasing hormone agonists with or without antiandrogen therapy. Patients were excluded if their clinicopathological data or follow-up information were incomplete. After exclusion, 630 patients were included in the final analysis. This study was approved by the Institutional Review Board of Kaohsiung Medical University Hospital (KMU-HIRB-2013132), and all participants provided written informed consent. The study was conducted in accordance with the guidelines of the Declaration of Helsinki and Good Clinical Practice. Baseline demographic and clinicopathological data, including age at ADT initiation, prostate-specific antigen (PSA) level, clinical stage, Gleason score, PSA nadir, and time to PSA nadir, were extracted from the medical records. Patients were routinely followed up at monthly visits, and PSA measurements were obtained every 3 months. Disease progression was defined as a confirmed biochemical recurrence, indicated by at least two consecutive increases in PSA levels exceeding the PSA nadir and separated by more than 1 week, or the initiation of secondary hormonal therapy for increasing PSA levels [16]. The time to progression was calculated from ADT initiation to the date of progression. Overall survival (OS) was defined as the time from ADT initiation to death from any cause, and prostate cancer-specific survival (CSS) was defined as the time from ADT initiation to death from prostate cancer. The causes of death were ascertained by referring to the National Death Registry maintained by the Ministry of Health and Welfare of Taiwan. Disease progression was observed in 518 patients during a median follow-up period of 165.8 months (Table S1). Four hundred and fourteen deaths were recorded, of which 314 were attributable to cancer [17]. Key clinical variables, including age, PSA level at ADT initiation, clinical stage, Gleason score, PSA nadir, and time to PSA nadir, were significantly associated with progression-free survival (PFS), OS, and CSS (*p* < 0.05).

### 2.3. Single-Nucleotide Polymorphism (SNP) Selection and Genotyping

SNPs were selected from the following 14 genes identified as commonly regulated by MEHP in transcriptomic analyses: zinc finger protein 433 (*ZNF433*), solute carrier family 25 member 47 (*SLC25A47*), asialoglycoprotein receptor 2 (*ASGR2*), endothelin 2 (*EDN2*), multiple EGF-like domains 6 (*MEGF6*), myelin protein zero–like 2 (*MPZL2*), ADAM metallopeptidase with thrombospondin type 1 motif 8 (*ADAMTS8*), EF-hand domain family member D2 (*EFHD2*), 3-hydroxyacyl-CoA dehydratase 4 (*HACD4*), secreted frizzled related protein 1 (*SFRP1*), N-Myc downstream regulated 1 (*NDRG1*), pyruvate dehydrogenase kinase 1 (*PDK1*), phosphodiesterase 5A (*PDE5A*), and anoctamin 4 (*ANO4*). Haplotype-tagged SNPs were identified using Haploview software (v4.2) based on linkage disequilibrium patterns in Han Chinese populations (Beijing and Southern Han Chinese) from the 1000 Genomes Project [18]. The selection criteria included a minor allele frequency (MAF) > 0.03 and a linkage disequilibrium (LD) threshold of *r*^2^ > 0.8. Genomic DNA was extracted from peripheral blood leukocytes using a QIAamp DNA Blood Kit (Qiagen, Hilden, Germany) according to the manufacturer’s instructions. Genotyping was performed at the National Center for Genome Medicine, Taiwan, using Axiom genotyping arrays (Affymetrix, Santa Clara, CA, USA) [17]. Standard quality control procedures were applied, excluding samples with low call rates and SNPs with call rates < 90%, MAF < 0.03, or deviations from Hardy–Weinberg equilibrium (*p* < 0.0001). After quality control filtering, 158 SNPs were retained for subsequent analyses. The genotyping concordance among blinded duplicate samples was 100%.

### 2.4. Bioinformatic Analyses of ANO4 Expression and Function

To evaluate the clinical relevance of *ANO4* in prostate cancer, gene expression data were obtained from multiple public resources, including PCaDB [19], the Gene Expression Database of Normal and Tumor Tissues 2 [20], and The Cancer Genome Atlas Prostate Adenocarcinoma (TCGA-PRAD) dataset. Differences in *ANO4* expression levels between prostate cancer and normal prostate tissues were assessed using pooled analyses with a random-effects model implemented in Review Manager (v5.4.1; Cochrane, London, UK). For mechanistic insights, TCGA-PRAD samples were stratified into high- and low-expression groups based on the median *ANO4* expression. Differential gene expression analysis was performed using the Limma package (v3.64.1). The ranked gene lists were subjected to GSEA for GO and KEGG gene sets from the Molecular Signatures Database. Enrichment significance was determined using 1000 permutations, with a false discovery rate (FDR) < 0.05, which was considered statistically significant. Key enriched pathways were visualized using the Enrichplot package (v1.28.2).

### 2.5. Statistical Analyses

Statistical analyses were performed using R and SPSS software (v19.0; IBM, Armonk, NY, USA). Associations among genetic variants, clinicopathological variables, and clinical outcomes were evaluated using Cox proportional hazards regression models. Hazard ratios (HRs) and 95% confidence intervals (CIs) were calculated. For this exploratory genetic association study, initial screening was conducted using a nominal two-sided *p*-value < 0.05. To account for multiple testing, FDR *q*-values were calculated; candidate genes were then prioritized based on the convergence of evidence across various clinical endpoints.

## 3. Results

**Figure 1:**
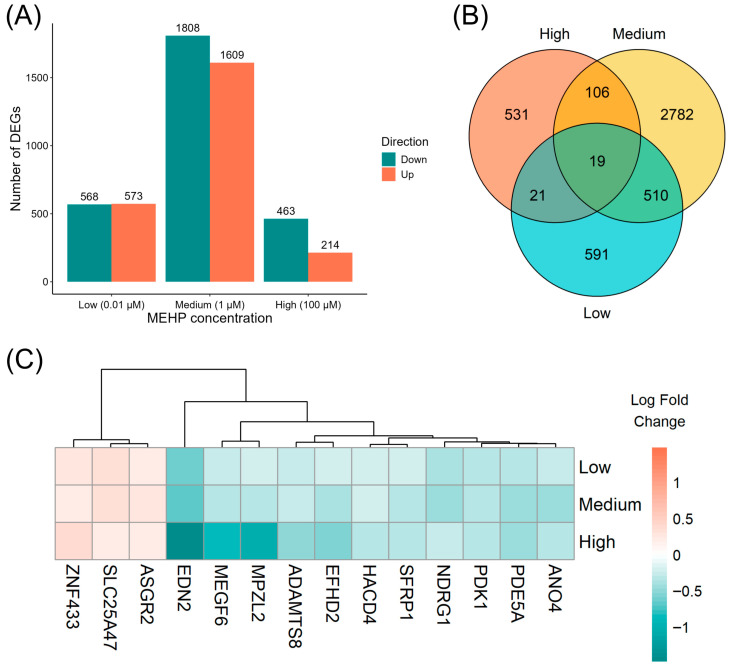
The transcriptomic landscape of mono-2-ethylhexyl phthalate (MEHP)-induced alterations in human prostate cells. (**A**) Number and direction of change in differentially expressed genes (DEGs; *p* < 0.05) following the treatment of human immortalized normal prostatic epithelial (PNT1A) cells with vehicle control, or MEHP at 0.01 μM, 1 μM, or 100 μM. (**B**) Venn diagram illustrating the overlap of DEGs across MEHP concentration groups. (**C**) Heatmap showing the log fold changes in 14 shared DEGs with concordant directionality across all MEHP concentrations. Orange indicates upregulation and green indicates downregulation relative to dimethyl sulfoxide-treated controls.

To characterize the transcriptional effects of MEHP in prostate cells, human immortalized normal prostatic epithelial (PNT1A) cells were exposed to vehicle control, low (0.01 μM), medium (1 μM), or high (100 μM) concentrations of MEHP. Differential expression analysis identified a substantial number of MEHP-responsive genes across all exposure levels (*p* < 0.05; Figure 1A). Specifically, 1141, 3417, and 677 differentially expressed genes (DEGs) were detected following low-, medium-, and high-dose MEHP treatments, respectively. Because strict FDR control yielded insufficient DEGs for robust pathway analysis, we employed a tiered approach to prioritize the most reliable targets. Comparative analysis revealed 19 DEGs that were consistently altered at all three MEHP concentrations (Figure 1B). Among these shared genes, 14 exhibited concordant directional changes, including three upregulated and 11 downregulated genes. Furthermore, seven of these genes demonstrated clear dose-dependent expression patterns with increasing MEHP concentration (Figure 1C), suggesting robust and biologically relevant transcriptional responses to MEHP exposure.

**Figure 2:**
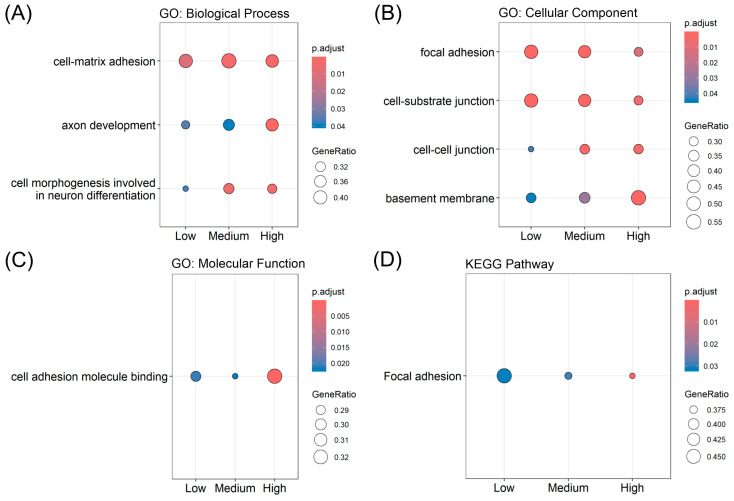
Functional enrichment analysis of MEHP-responsive genes in human prostate cells. Gene set enrichment analysis of shared Gene Ontology (GO) terms following MEHP treatment, categorized by (**A**) biological processes, (**B**) cellular components, and (**C**) molecular functions. (**D**) Kyoto Encyclopedia of Genes and Genomes (KEGG) pathway enrichment analysis highlighting the shared pathways that were significantly affected by MEHP exposure.

To investigate the biological significance of the MEHP-induced transcriptional changes, GO, KEGG, and GSEA were performed using ranked differential expression profiles. GSEA revealed a significant negative enrichment of biological processes related to cell–matrix adhesion, axon development, and cell morphogenesis involved in neuron differentiation across all MEHP concentrations (Figure 2A). Consistent with this, cellular component analysis showed negative enrichment in focal adhesions, cell–substrate junctions, cell–cell junctions, and basement membranes (Figure 2B). Molecular function analysis further highlighted the binding of cell adhesion molecules (Figure 2C). Corroborating these findings, the KEGG pathway analysis identified focal adhesion as a significantly downregulated pathway following MEHP treatment (Figure 2D), underscoring the potential role of MEHP in impairing prostate epithelial cell adhesion and structural integrity.

**Figure 3:**
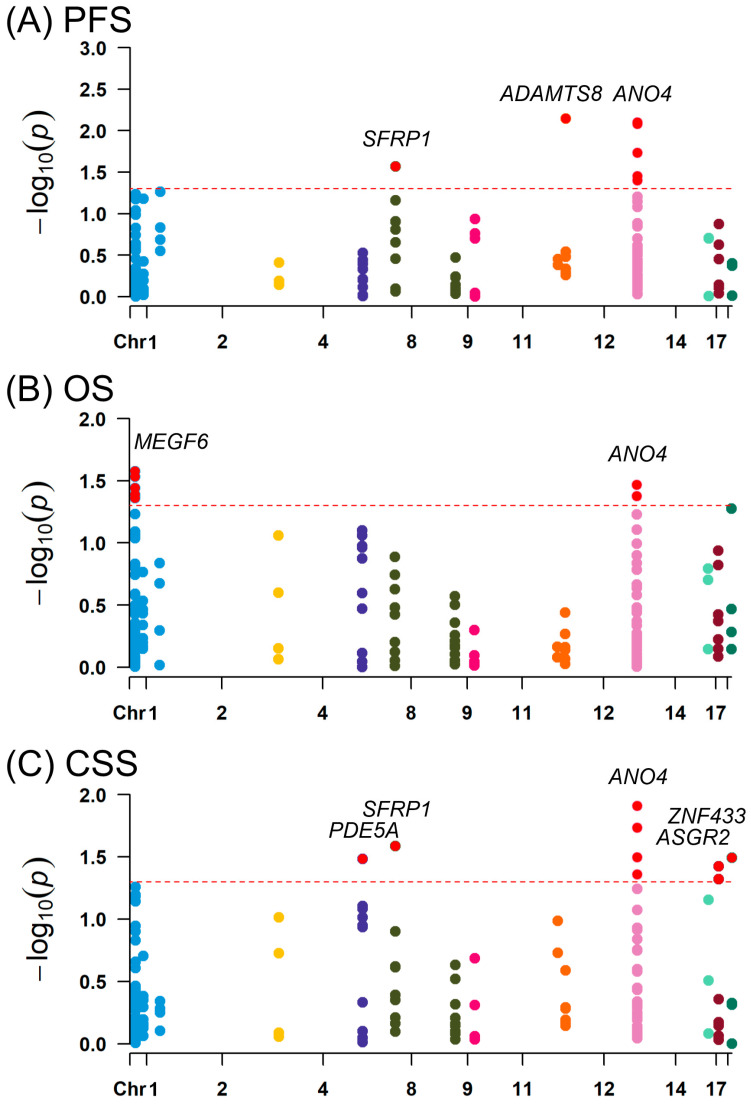
Association of single nucleotide polymorphisms (SNPs) in MEHP-regulated genes with clinical outcomes in patients with prostate cancer. Manhattan plots depicting the associations between 158 SNPs in 14 MEHP-regulated genes and (**A**) progression-free survival, (**B**) overall survival, and (**C**) cancer-specific survival in patients with prostate cancer receiving androgen deprivation therapy. The y-axis represents −log10(*p*) values, and the x-axis represents the chromosomal position of the SNPs. The distinct colors of the circles along the x-axis are used to visually differentiate the SNPs grouped by their respective chromosomes. The dashed red line denotes the significance threshold of *p* = 0.05. Significant SNPs are highlighted with red circles.

To explore the clinical relevance of MEHP-responsive genes in prostate cancer progression, we evaluated 158 SNPs in 14 common MEHP-regulated genes in a cohort of patients with prostate cancer treated with ADT. Our initial exploratory analysis identified distinct genetic determinants of prognosis at a nominal *p* < 0.05 (Figure 3). Specifically, seven SNPs in *SFRP1*, *ADAMTS8*, and *ANO4* were significantly associated with PFS, while seven SNPs in *MEGF6* and *ANO4* were associated with OS. Furthermore, nine SNPs in *PDE5A*, *SFRP1*, *ANO4*, *ASGR2*, and *ZNF433* were significantly associated with CSS. Among them, rs17485225 in *ANO4* was significantly associated with both OS and CSS. Carriers of the minor A allele of rs17485225 exhibited a reduced risk of all-cause mortality (HR = 0.78, 95% CI = 0.63–0.98, *p* = 0.034) and cancer-specific mortality (HR = 0.76, 95% CI = 0.59–0.99, *p* = 0.044) compared with carriers of the major G allele. While the calculated FDR for individual SNPs such as rs17485225 was high (*q* ≥ 0.706), *ANO4* emerged as the only gene harboring SNPs that were associated with all three clinical endpoints, providing the converging evidence necessary to prioritize it as a primary candidate gene.

**Figure 4:**
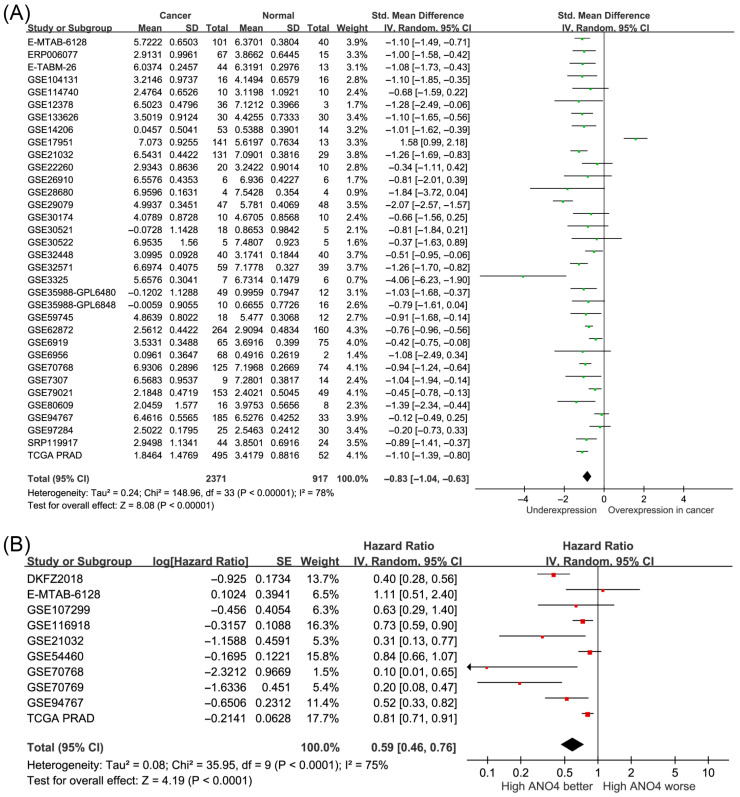
Clinical significance of *ANO4* expression in prostate cancer. (**A**) Pooled analysis of 34 public datasets showing significantly lower *ANO4* expression levels in prostate cancer tissues than in normal prostate tissues. (**B**) Pooled survival analysis demonstrating that a high *ANO4* expression level is associated with improved prognosis in patients with prostate cancer. The green squares represent the point estimates (standardized mean difference) for each individual study in (A), and the red squares represent the point estimates (hazard ratio) in (B), with the size of the square proportional to the study’s statistical weight. The horizontal lines denote the 95% confidence intervals (CIs) for each study, and arrows indicate where a CI exceeds the plotted x-axis range. The black diamond at the bottom represents the pooled overall effect size, with its width corresponding to the overall 95% CI. SD, standard deviation. SE, standard error. IV, inverse variance. Std, standardized. TCGA PRAD, The Cancer Genome Atlas Prostate Adenocarcinoma. df, degrees of freedom.

Given the consistent genetic associations observed for *ANO4*, we examined its expression and clinical relevance in prostate cancer. A pooled analysis of 34 public datasets, comprising 2371 prostate cancer samples and 917 normal prostate tissue samples, demonstrated that *ANO4* expression levels were significantly lower in prostate cancer tissues than in normal prostate tissues (standardized mean difference [SMD] = −0.83, 95% CI = −1.04 to −0.63, *p* < 0.001; Figure 4A). Although substantial statistical heterogeneity (*I*^2^ = 78%) indicates that the exact magnitude of the effect naturally varies across different clinical cohorts, the overarching directional trend consistently demonstrates reduced *ANO4* expression in cancer. Furthermore, pooled survival analysis of 10 independent studies revealed that higher *ANO4* expression levels were significantly associated with improved prognosis in patients with prostate cancer (HR = 0.59, 95% CI = 0.46–0.76, *p* < 0.001; Figure 4B), exhibiting a similarly consistent directional association despite cross-cohort variance (*I*^2^ = 75%). These findings support the potential tumor-suppressive role of *ANO4* in prostate cancer progression, which is compromised by MEHP exposure.

**Figure 5:**
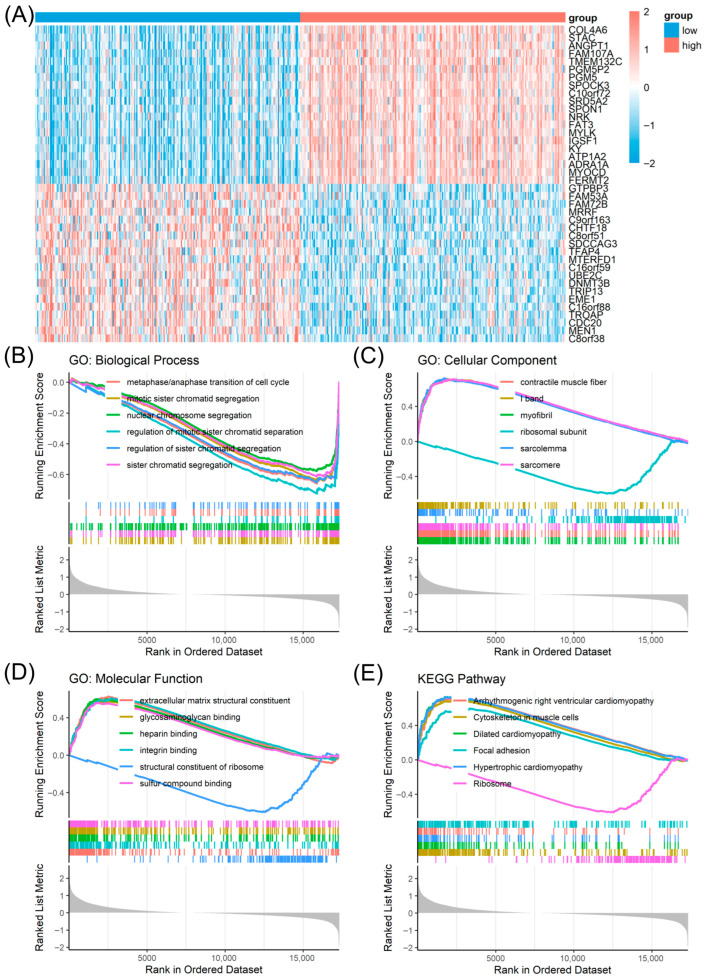
Functional characterization of genes associated with *ANO4* expression. (**A**) Heatmap displaying the top 20 upregulated and downregulated differentially expressed genes, distinguishing the high- and low-*ANO4* expression groups in The Cancer Genome Atlas Prostate Adenocarcinoma cohort. Gene set enrichment plots for genes correlated with *ANO4* expression, highlighting key pathways in (**B**) GO biological processes, (**C**) cellular components, (**D**) molecular functions, and (**E**) KEGG pathways.

To investigate the mechanism underlying the protective effect of *ANO4*, we compared the transcriptomes of high- and low-*ANO4* expression groups in the TCGA-PRAD dataset. GSEA revealed that the low-*ANO4* expression group, which had poorer survival outcomes, was significantly enriched in cell cycle-related processes, including chromosome segregation and sister chromatid separation (Figure 5B). Conversely, the high-*ANO4* expression group was positively enriched for structural integrity terms, including contractile muscle fibers, sarcomere organization, and extracellular matrix constituents, and negatively enriched for ribosomal subunit components (Figure 5C,D). In agreement with our MEHP treatment data, KEGG analysis of *ANO4*-correlated genes further highlighted focal adhesion and cytoskeleton-related pathways, while showing the downregulation of ribosome pathways (Figure 5E). These findings reinforce the hypothesis that MEHP-mediated suppression of *ANO4* compromises focal adhesion integrity and potentially drives disease progression.

## 4. Discussion

Our study integrates transcriptomic and clinical analyses of the effects of the plasticizer metabolite MEHP on human prostate epithelial cells, revealing potential gene–environment interactions contributing to prostate cancer progression. We demonstrated that MEHP exposure induced marked transcriptional reprogramming, predominantly characterized by the downregulation of genes involved in focal adhesion and cell–matrix interactions. Importantly, genetic variants within MEHP-responsive genes, notably *ANO4*, were significantly associated with survival outcomes in patients undergoing ADT. Reduced *ANO4* expression, recapitulating the suppressive effects of MEHP, correlated with poor prognosis. These observations suggest a proposed gene–environment model wherein *ANO4* may serve as a potential mediator associating environmental exposure with dysregulated cell cycle control and compromised focal adhesion integrity in prostate cancer, although our current data cannot establish definitive causality.

Accumulating evidence indicates that MEHP promotes cancer progression by activating pro-migratory and invasive signaling. In testicular embryonal carcinoma models, MEHP increases matrix metalloproteinase expression and MYC activation, thereby enhancing extracellular matrix degradation and invasion; this aligns with our observation that MEHP suppresses cell–matrix adhesion genes and disrupts structural integrity [21]. Additionally, MEHP activates non-canonical oncogenic pathways, including G protein-coupled estrogen receptor-mediated AKT signaling, transforming growth factor-β/SMAD signaling, and nuclear factor-κB signaling, which collectively enhance survival, migration, and invasion across multiple cancers [22,23,24]. Unlike previous studies emphasizing signaling activation, our data uniquely identified focal adhesion suppression as the central mechanism by which MEHP may facilitate malignant progression. Focal adhesions anchor epithelial cells to the extracellular matrix and transduce inhibitory cues restraining motility and proliferation. Destabilizing these complexes compromises adhesion strength, facilitates cytoskeletal remodeling, and enhances migratory and metastatic potential. Moreover, this destabilization promotes resistance to anoikis (apoptosis induced by matrix detachment), enabling the survival of disseminating tumor cells [25]. Collectively, the observed MEHP-associated suppression of focal adhesions may cooperate with diverse oncogenic signals to facilitate prostate cancer progression.

Consistent with the tumor-promoting effects of MEHP, our clinical and bioinformatics analyses identified *ANO4* as a protective gene; its downregulation predicted adverse prostate cancer outcomes. Although initially characterized as a TMEM16 family member with limited Ca^2+^-activated chloride channel activity and modest proliferative effects in select cellular contexts [26], *ANO4* increasingly demonstrates broader roles in the regulation of plasma membrane dynamics linked to cell adhesion and motility. Notably, *ANO4* exhibits phospholipid scramblase activity, promotes phosphatidylserine externalization, and enhances the sheddase activity of ADAM family metalloproteases, including ADAM10 and ADAM17. This modulates the cleavage of adhesion receptors and growth factor precursors, facilitating controlled adhesion remodeling rather than unchecked invasion [27]. In non-metastatic clear cell renal cell carcinoma, reduced *ANO4* expression associates with advanced stage and poor survival, while epithelial adhesion and junction integrity pathways are enriched in tumors with high *ANO4* expression [28]. Furthermore, specific *ANO4* germline variants, notably rs585335, independently predict biochemical recurrence following radical prostatectomy [29], suggesting host genetics may influence susceptibility to MEHP-induced *ANO4* suppression and subsequent tumor progression.

To explore the functional relevance of the *ANO4* variants, we performed in silico functional annotations via the HaploReg database. The intronic SNP rs17485225 likely exerts its effects through LD with regulatory elements rather than directly altering protein structure. Our analysis revealed that rs17485225 is in strong LD (*r*^2^ > 0.9) with neighboring variants situated within regions enriched for active enhancer marks and DNase hypersensitivity sites. These linked variants are predicted to alter binding motifs of transcription factors critical for cell cycle regulation and oncogenesis, such as AP-1, GATA, STAT, and p53, and demonstrate established binding to transcriptional coactivators like P300. Although queried databases lacked significant expression quantitative trait loci directly linking this haplotype to *ANO4* expression in available target tissues, the convergence of strong enhancer chromatin states and disrupted transcription factor motifs strongly suggests a gene regulatory role. Consequently, we hypothesize that these non-coding variants may modulate *ANO4* transcriptional activity in response to environmental stimuli or hormonal shifts, though direct experimental validation via reporter assays or genome editing in prostate cancer models is required.

Our pathway analyses further delineated the downstream consequences of *ANO4* downregulation, revealing a transcriptional program characterized by upregulated cell cycle and ribosome biogenesis pathways alongside suppressed focal adhesion signaling. Activating cell cycle regulators drives uncontrolled proliferation [30], while increased ribosome biogenesis supports the heightened protein synthesis demands of rapidly dividing tumor cells [31]. Conversely, intact focal adhesion signaling restrains metastasis by preserving epithelial integrity and contact inhibition [32]. Accumulating evidence supports a biphasic model wherein excessive upregulation and stabilization of focal adhesion machinery suppress, rather than promote, cancer progression. Although dynamic turnover is required for efficient migration, excessive reinforcement of focal adhesion complexes mechanically anchors tumor cells to the extracellular matrix, thereby impairing motility, mitotic rounding, and invasion [33]. Upregulation of adhesion-stabilizing factors, including deleted in liver cancer 1, tensin 2, and integrin α2β1, shifts cells toward a strong-adhesion state characterized by large, super-mature focal adhesions with low turnover rates [34,35,36]. Thus, *ANO4* loss, either intrinsically or through MEHP exposure, may undermine this protective axis, potentially converging enhanced proliferation with increased metastatic potential. This dual effect offers a hypothetical mechanistic framework associating environmental toxicant exposure with aggressive prostate cancer phenotypes, which requires validation in cohorts with matched exposure and genomic data.

The strengths of this study include integrating in vitro transcriptomic profiling with clinical genetic association analyses in a well-characterized cohort of ADT-treated patients. This multidimensional approach supports a biologically plausible link among MEHP exposure, host genetics, and clinical outcomes. Nevertheless, several limitations warrant consideration. First, although utilizing immortalized normal prostatic epithelial PNT1A cells modeled early field cancerization, this non-tumorigenic model cannot fully recapitulate established prostate cancer biology. Specifically, it lacks intra-tumoral heterogeneity, microenvironmental interactions, and the unique selective pressures of the androgen-deprived state relevant to our clinical cohort. Although we partially bridged this gap by validating *ANO4* in the TCGA-PRAD dataset, future mechanistic studies must employ tumor-derived models under androgen deprivation to validate the MEHP-*ANO4* axis. Second, the functional consequences of the identified *ANO4* SNPs on expression and protein activity require experimental validation. Third, to avoid prematurely eliminating vital biological signals in this exploratory risk-gene hunting study, our genetic screening utilized nominal *p*-values without strict FDR correction. We mitigated false positives by prioritizing *ANO4* based on converging evidence across all three clinical endpoints; nonetheless, these exploratory signals require independent validation. Finally, our exposure inference relies solely on in vitro transcriptomic perturbation combined with independent clinical genetic associations. Because our retrospective design lacks patient-level phthalate measurements, exposure and genotype were never linked within the same individuals. Consequently, causality between MEHP exposure and clinical outcomes cannot be established. This underscores the need for prospective studies incorporating paired phthalate metabolite profiling alongside genomic data to validate these proposed gene–environment associations.

## 5. Conclusions

In conclusion, our findings implicate MEHP as a potential modulator of prostate epithelial adhesion programs, identifying focal adhesion suppression and reduced *ANO4* expression as factors associated with aggressive prostate cancer progression. This work highlights the importance of gene–environment interactions in cancer biology, positioning focal adhesion networks as potential biomarkers or therapeutic targets. Future in vivo studies of MEHP exposure and functional dissection of *ANO4*-mediated adhesion signaling are essential to validate these mechanisms and assess their translational relevance.

## Data Availability

The genetic data for our clinical cohort are not publicly available due to privacy and ethical restrictions. The transcriptomic data analyzed in this study are publicly available from the Gene Expression Omnibus (GSE67396 and GSE67397) and the TCGA data portal.

## Supplementary Materials

The following supporting information can be downloaded at: https://www.mdpi.com/article/10.3390/diagnostics16050794/s1, Table S1. Clinical and pathological characteristics of the study population.

## References

[1] Jaeger R.J., Rubin R.J. (1973). Extraction, localization, and metabolism of di-2-ethylhexyl phthalate from PVC plastic medical devices. Environ. Health Perspect..

[2] Ashaari S., Jamialahmadi T., Davies N.M., Almahmeed W., Sahebkar A. (2025). Di (2-ethyl hexyl) phthalate and its metabolite-induced metabolic syndrome: A review of molecular mechanisms. Drug Chem. Toxicol..

[3] Li X., Xiao C., Liu J., Wei N., Song J., Yuan J., Liu L., Song R., Yi W., Pan R. (2024). Association of Di(2-ethylhexyl) Phthalate Exposure with Reproductive Hormones in the General Population and the Susceptible Population: A Systematic Review and Meta-Analysis. Environ. Health.

[4] Zheng W.C., Xu W.T., Chen C.R., Lin F., Chen S.H., Li X.D., Sun X.L., Zheng Q.S., Wei Y., Xue X.Y. (2026). Integrative analysis of DEHP exposure and prostate cancer: Mechanistic insights and predictive modeling. Environ. Pollut..

[5] Martinez-Razo L.D., Rivero-Segura N.A., Almeida-Aguirre E.K.P., Mancilla-Herrera I., Rincon-Heredia R., Martinez-Ibarra A., Cerbon M. (2025). Mono(2-ethylhexyl) Phthalate Disrupts Mitochondrial Function, Dynamics and Biogenesis in Human Trophoblast Cells at Human Exposure Range Concentrations. Toxics.

[6] Rusyn I., Corton J.C. (2012). Mechanistic considerations for human relevance of cancer hazard of di(2-ethylhexyl) phthalate. Mutat. Res..

[7] Wu J.H., Chen J., Wang Y., Xia B., Wang R., Zhao Y., Wang Q.X., Song Q., Yao S.H., Zhang Y.H. (2017). Effect of Mono-2-ethyhexyl Phthalate on DNA Methylation in Human Prostate Cancer LNCaP Cells. Biomed. Environ. Sci..

[8] Wu P.H., Chen S.C., Chien C.J., Lin J., Lee H.Y., Lin Y.T., Weng T.C., Hsu P.C., Wu M.T., Yu S.H. (2025). Epigenetic signatures of phthalate exposure and potential risks: A DNA methylation analysis using Infinium MethylationEPIC BeadChip. Environ. Epigenetics.

[9] Wu A.H., Franke A.A., Wilkens L.R., Tseng C., Conroy S.M., Li Y., Polfus L.M., De Rouen M., Caberto C., Haiman C. (2021). Urinary phthalate exposures and risk of breast cancer: The Multiethnic Cohort study. Breast Cancer Res..

[10] Zhou F., Guo C., Wang L., Zhang G., Wang J., Chen W., Cui K., Tan Y., Zhou Z. (2023). Mono-(2-ethylhexyl) Phthalate (MEHP)-Induced Telomere Structure and Function Disorder Mediates Cell Cycle Dysregulation and Apoptosis via c-Myc and Its Upstream Transcription Factors in a Mouse Spermatogonia-Derived (GC-1) Cell Line. Toxics.

[11] Dhaini H.R., Daher Z. (2019). Genetic polymorphisms of PPAR genes and human cancers: Evidence for gene-environment interactions. J. Environ. Sci. Health C Environ. Carcinog. Ecotoxicol. Rev..

[12] Ke C.C., Chen L.C., Yu C.C., Cheng W.C., Huang C.Y., Lin V.C., Lu T.L., Huang S.P., Bao B.Y. (2020). Genetic Analysis Reveals a Significant Contribution of CES1 to Prostate Cancer Progression in Taiwanese Men. Cancers.

[13] Liu J.C., Shen W.C., Shih T.C., Tsai C.W., Chang W.S., Cho Y., Tsai C.H., Bau D.T. (2015). The current progress and future prospects of personalized radiogenomic cancer study. Biomedicine.

[14] Subramanian A., Tamayo P., Mootha V.K., Mukherjee S., Ebert B.L., Gillette M.A., Paulovich A., Pomeroy S.L., Golub T.R., Lander E.S. (2005). Gene set enrichment analysis: A knowledge-based approach for interpreting genome-wide expression profiles. Proc. Natl. Acad. Sci. USA.

[15] Bao B.Y., Pao J.B., Huang C.N., Pu Y.S., Chang T.Y., Lan Y.H., Lu T.L., Lee H.Z., Juang S.H., Chen L.M. (2011). Polymorphisms inside microRNAs and microRNA target sites predict clinical outcomes in prostate cancer patients receiving androgen-deprivation therapy. Clin. Cancer Res..

[16] Ross R.W., Oh W.K., Xie W., Pomerantz M., Nakabayashi M., Sartor O., Taplin M.E., Regan M.M., Kantoff P.W., Freedman M. (2008). Inherited variation in the androgen pathway is associated with the efficacy of androgen-deprivation therapy in men with prostate cancer. J. Clin. Oncol..

[17] Chang H.H., Lee C.H., Chen Y.T., Huang C.Y., Yu C.C., Lin V.C., Geng J.H., Lu T.L., Huang S.P., Bao B.Y. (2022). Genetic Analysis Reveals the Prognostic Significance of the DNA Mismatch Repair Gene MSH2 in Advanced Prostate Cancer. Cancers.

[18] Barrett J.C., Fry B., Maller J., Daly M.J. (2005). Haploview: Analysis and visualization of LD and haplotype maps. Bioinformatics.

[19] Li R., Zhu J., Zhong W.-D., Jia Z. (2022). PCaDB—A comprehensive and interactive database for transcriptomes from prostate cancer population cohorts. bioRxiv.

[20] Park S.J., Yoon B.H., Kim S.K., Kim S.Y. (2019). GENT2: An updated gene expression database for normal and tumor tissues. BMC Med. Genom..

[21] Yao P.L., Lin Y.C., Richburg J.H. (2012). Mono-(2-ethylhexyl) phthalate (MEHP) promotes invasion and migration of human testicular embryonal carcinoma cells. Biol. Reprod..

[22] Fan P., Li Z., Zuo C., Fang M. (2020). Promotion effects of mono-2-ethyhexyl phthalate (MEHP) on migration and invasion of human melanoma cells via activation of TGF-β signals. Cell Biochem. Funct..

[23] Wang Z., Shao M., Liu Y. (2017). Promotion of Wilms’ tumor cells migration and invasion by mono-2-ethyhexyl phthalate (MEHP) via activation of NF-κB signals. Chem. Biol. Interact..

[24] Yang W., Tan W., Zheng J., Zhang B., Li H., Li X. (2018). MEHP promotes the proliferation of cervical cancer via GPER mediated activation of Akt. Eur. J. Pharmacol..

[25] Liu B., Wu Q., Xuan Z., Zheng Z., Du Y., Sui X., Wu H., Zhang Z., Zhang Z., Zhong M. (2025). Mechanisms Involved in Focal Adhesion Signaling Regulating Tumor Anoikis Resistance. Cancer Sci..

[26] Maniero C., Scudieri P., Haris Shaikh L., Zhao W., Gurnell M., Galietta L.J.V., Brown M.J. (2019). ANO4 (Anoctamin 4) Is a Novel Marker of Zona Glomerulosa That Regulates Stimulated Aldosterone Secretion. Hypertension.

[27] Leitzke S., Seidel J., Ahrens B., Schreiber R., Kunzelmann K., Sperrhacke M., Bhakdi S., Reiss K. (2022). Influence of Anoctamin-4 and -9 on ADAM10 and ADAM17 Sheddase Function. Membranes.

[28] Al Sharie A.H., Al Zu’bi Y.O., El-Elimat T., Al-Kammash K., Abu Lil A., Isawi I.H., Al Sharie S., Abu Mousa B.M., Al Malkawi A.A., Alali F.Q. (2023). ANO4 Expression Is a Potential Prognostic Biomarker in Non-Metastasized Clear Cell Renal Cell Carcinoma. J. Pers. Med..

[29] Yu C.C., Chen L.C., Huang C.Y., Lin V.C., Lu T.L., Lee C.H., Huang S.P., Bao B.Y. (2020). Genetic association analysis identifies a role for ANO5 in prostate cancer progression. Cancer Med..

[30] Cavalu S., Abdelhamid A.M., Saber S., Elmorsy E.A., Hamad R.S., Abdel-Reheim M.A., Yahya G., Salama M.M. (2024). Cell cycle machinery in oncology: A comprehensive review of therapeutic targets. FASEB J..

[31] Hwang S.P., Denicourt C. (2024). The impact of ribosome biogenesis in cancer: From proliferation to metastasis. NAR Cancer.

[32] Pang X., He X., Qiu Z., Zhang H., Xie R., Liu Z., Gu Y., Zhao N., Xiang Q., Cui Y. (2023). Targeting integrin pathways: Mechanisms and advances in therapy. Signal Transduct. Target. Ther..

[33] Liu Z., Zhang X., Ben T., Li M., Jin Y., Wang T., Song Y. (2025). Focal adhesion in the tumour metastasis: From molecular mechanisms to therapeutic targets. Biomark. Res..

[34] Hong S.Y., Shih Y.P., Sun P., Hsieh W.J., Lin W.C., Lo S.H. (2016). Down-regulation of tensin2 enhances tumorigenicity and is associated with a variety of cancers. Oncotarget.

[35] Ramirez N.E., Zhang Z., Madamanchi A., Boyd K.L., O’Rear L.D., Nashabi A., Li Z., Dupont W.D., Zijlstra A., Zutter M.M. (2011). The α_2_β_1_ integrin is a metastasis suppressor in mouse models and human cancer. J. Clin. Investig..

[36] Shih Y.P., Takada Y., Lo S.H. (2012). Silencing of DLC1 upregulates PAI-1 expression and reduces migration in normal prostate cells. Mol. Cancer Res..

