# Supplementary material for: From Plastics to Prognosis: ANO4 Susceptibility Links Phthalate Exposure to Prostate Cancer Progression
Source: Diagnostics (Basel). 2026 Mar 7;16(5):794. doi: 10.3390/diagnostics16050794 (PMC12985240; doi:10.3390/diagnostics16050794)
Supplement: Supplementary file 1 [file diagnostics-16-00794-s001.zip › diagnostics-4154743-supplementary.pdf]

Table S1. Clinical and pathological characteristics of the study population

Characteristics	n (%)	PFS		OS		CSS	
		HR (95%CI)	*p*	HR (95%CI)	*p*	HR (95%CI)	*p*
Total, n (%)	630	518 (82.6)		414 (65.7)		314 (49.8)	
Age at diagnosis, years							
Median (IQR)	73 (67-79)						
<74	344 (54.7)	1.00		1.00		1.00	
≥74	285 (45.3)	0.72 (0.61-0.86)	<0.001	1.67 (1.37-2.03)	<0.001	1.32 (1.06-1.65)	0.014
PSA at ADT initiation, ng/mL							
Median (IQR)	34.5 (11.25-129)						
<35	307 (50.6)	1.00		1.00		1.00	
≥35	300 (49.4)	1.21 (1.02-1.45)	0.030	1.99 (1.63-2.44)	<0.001	2.31 (1.83-2.91)	<0.001
Clinical stage at diagnosis							
T1/T2	187 (29.9)	1.00		1.00		1.00	
T3/T4/N1	205 (32.8)	0.86 (0.69-1.08)	0.190	1.07 (0.82-1.39)	0.642	1.08 (0.79-1.49)	0.636
M1	233 (37.3)	1.41 (1.14-1.74)	0.002	2.51 (1.97-3.19)	<0.001	3.10 (2.33-4.11)	<0.001
Gleason score at diagnosis							
2-6	188 (30.6)	1.00		1.00		1.00	
7	194 (31.6)	1.16 (0.93-1.46)	0.189	1.06 (0.82-1.38)	0.640	1.06 (0.78-1.44)	0.694
8-10	232 (37.8)	1.48 (1.19-1.84)	<0.001	1.96 (1.54-2.48)	<0.001	2.14 (1.63-2.82)	<0.001
PSA nadir, ng/mL							
Median (IQR)	0.14 (0.01-1.16)						
<0.15	314 (50.7)	1.00		1.00		1.00	
≥0.15	305 (49.3)	2.48 (2.07-2.96)	<0.001	2.95 (2.41-3.61)	<0.001	3.57 (2.82-4.53)	<0.001
Time to PSA nadir, months							

Median (IQR)	11 (5-20)						
<12	323 (52.2)	1.00		1.00		1.00	
≥12	296 (47.8)	0.36 (0.31-0.44)	<0.001	0.66 (0.54-0.80)	<0.001	0.57 (0.45-0.71)	<0.001

Abbreviations: PFS, progression-free survival; OS, overall survival; CSS, cancer-specific survival; IQR, interquartile range; PSA, prostate-specific antigen; ADT, androgen deprivation therapy.

Median follow-up duration was 165.8 months.

Totals may not sum to the overall number of patients because of missing data.
